# Padronizando a Exposição à Radiação durante o Cateterismo Cardíaco em Crianças com Cardiopatia Congênita: Dados de um Registro Multicêntrico Brasileiro

**DOI:** 10.36660/abc.20190012

**Published:** 2020-12-01

**Authors:** João Luiz Manica, Vanessa Oliveira Duarte, Marcelo Ribeiro, Adam Hartley, Ricardo Petraco, Carlos Pedra, Raul Rossi

**Affiliations:** 1 Instituto de Cardiologia Fundação Universitária de Cardiologia Porto AlegreRS Brasil Instituto de Cardiologia / Fundação Universitária de Cardiologia (IC/FUC),Porto Alegre, RS - Brasil; 2 Hospital do Coração São PauloSP Brasil Hospital do Coração, São Paulo, SP - Brasil; 3 National Heart and Lung Institute Imperial College London Hammersmith Hospital Londres Reino Unido National Heart and Lung Institute, Imperial College London, Hammersmith Hospital, Londres - Reino Unido

**Keywords:** Diagnóstico por Imagem/métodos, Vias de Exposição de Radiação, Cardiopatias Congênitas, Cateterismo Cardíaco/métodos, Criança

## Abstract

**Fundamento:**

Nos últimos anos, o recente aumento no número de procedimentos intervencionistas tem resultado em crescente preocupação em relação à exposição radiológica por pacientes e equipe médica. A avaliação da exposição dos níveis de radiação em crianças é difícil devido à grande variabilidade no peso corporal. Portanto, os valores de referência de radiação não estão bem definidos para essa população.

**Objetivos:**

Avaliar e validar a razão do produto dose-área (DAP) em relação ao peso corporal como uma medida de referência de radiação em cateterismos cardíacos em crianças.

**Métodos:**

Estudo multicêntrico observacional com dados do Registro Brasileiro de Cateterismo Cardíaco em Cardiopatias Congênitas (CHAIN) de março de 2013 a junho de 2014. Os critérios de inclusão foram: pacientes <18 anos submetidos a procedimentos hemodinâmicos para cardiopatia congênita, com DAP devidamente registrado. Foram considerados diferenças estatísticas significativas os valores de p < 0,05.

**Resultados:**

Este estudo avaliou 429 pacientes com idade e peso medianos de 50 (10, 103) meses e 15 (7, 28) kg, respectivamente. O DAP mediano foi de 742,2 (288,8, 1.791,5) μGy.m2. Houve uma boa correlação entre o DAP e o produto peso/tempo de fluoroscopia (rs=0,66). Não foi observada diferença estatisticamente significativa na relação DAP/peso entre procedimentos terapêuticos e diagnósticos. Houve ampla variação da relação DAP/peso entre os procedimentos terapêuticos (p<0.001).

**Conclusões:**

A proporção DAP/peso é a medida mais simples e aplicável para avaliar a exposição radiológica em uma população pediátrica. Apesar da escassa literatura disponível, as doses obtidas no presente estudo foram semelhantes àquelas encontradas anteriormente. Estudos de validação e comparação são importantes na avaliação do impacto de estratégias para redução da exposição radiológica nessa população. (Arq Bras Cardiol. 2020; [online].ahead print, PP.0-0)

## Introdução

Nos últimos 20 anos, o cateterismo cardíaco não só foi utilizado como exame diagnóstico de cardiopatias congênitas, mas também desempenhou um papel importante nos tratamentos paliativo e definitivo de mais de 50% dos pacientes com cardiopatias congênitas.^1^ Nesse período, a complexidade, a duração e o número de procedimentos percutâneos aumentaram, além de um consequente aumento da exposição dos pacientes à radiação ionizante.^2 - 4^

As crianças são altamente sensíveis à radiação ionizante, devido à maior proporção de células em divisão ativa e à grande fração da área corporal exposta.^2^ Assim, existe uma grande preocupação com os efeitos cumulativos, particularmente o alto risco de malignidade causado por danos cromossômicos a longo prazo, com relatos demonstrando que crianças são até dez vezes mais suscetíveis ao desenvolvimento de câncer por exposição à radiação do que adultos.^5 , 6^ Além disso, a dose de radiação efetiva é maior para crianças, resultando em uma dose de radiação mais alta para os órgãos vizinhos quando uma área de interesse está sendo avaliada.

Há poucos estudos sobre doses de radiação emitidas durante intervenções em crianças com cardiopatia congênita.^3 , 7^ Para obter redução da dose de radiação, é essencial estabelecer doses de referência que permitam comparações entre procedimentos.^4^ No entanto, é difícil avaliar a exposição à radiação em uma população pediátrica devido às diferenças nas complexidades dos procedimentos, idade e peso dos pacientes, bem como nos tipos de equipamentos utilizados.^8^ Além disso, o cálculo da dose efetiva estimada de radiação é complexo. Atualmente, a dose total de radiação (kerma no ar total) e o produto dose-área total ( *dose-area product* – DAP), que é a melhor forma de estimar os efeitos estocásticos (efeitos de radiação a longo prazo e risco de malignidade) e efeitos cumulativos da exposição, são utilizados como indicadores de uma dose cumulativa de radiação na pele.

Recentemente, Chida et al.^2^ e Kobayashi et al.^8^ observaram correlação entre DAP e peso como referência de dose de radiação em crianças. Eles concluíram que a dose de radiação tende a variar proporcionalmente ao tamanho do paciente. Nesse contexto, o presente estudo tem como objetivo avaliar a razão DAP/peso como referência de exposição a radiação em procedimentos de cateterismo cardíaco pediátrico realizados no Brasil.

## Materiais e Métodos

### Desenho e População do Estudo

Trata-se de um estudo observacional transversal em que pacientes com <18 anos de idade e participantes do registro de Intervenção e Angiografia de Cardiopatias Congênitas ( *Congenital Heart Disease Intervention and Angiography* – CHAIN), um registro brasileiro de cateterismo cardíaco para cardiopatia congênita, foram avaliados após um diagnóstico ou procedimento de intervenção entre 5 de março de 2013 e 30 de junho de 2014.

O registro CHAIN é um estudo prospectivo nacional multicêntrico, coordenado pelo Instituto de Ensino e Pesquisa do Hospital do Coração, em conjunto com o Ministério da Saúde e a Sociedade Brasileira de Hemodinâmica e Cardiologia Intervencionista. O principal objetivo foi reunir dados prospectivos e criar um registro nacional de cateterismo de pacientes com cardiopatias congênitas, além de propor uma análise abrangente da situação atual e elaborar medidas efetivas de ação para a saúde pública no Brasil.

Pacientes submetidos a procedimentos eletrofisiológicos ou aqueles em que o acesso vascular foi obtido por meio de procedimentos híbridos foram excluídos do estudo. Pacientes submetidos a mais de um cateterismo em datas diferentes foram considerados pacientes distintos em cada procedimento e incluídos nas estatísticas gerais, bem como no grupo de cada procedimento específico. Os pacientes submetidos a mais de uma intervenção utilizando o mesmo procedimento foram classificados de acordo com o procedimento mais complexo.

### Variáveis Analisadas

Características demográficas dos pacientes, como idade, sexo, peso, superfície corporal, tipo de cardiopatia e lesões residuais, foram obtidas através do registro CHAIN, além de dados referentes ao procedimento hemodinâmico realizado, incluindo tempo fluoroscópico e dose de exposição à radiação. O DAP, que representa a dose de radiação medida no ar em relação à distância do tubo de raios-X multiplicada pela área do feixe de raios-X a essa distância, foi expresso em μGy.m^2^. As medidas de radiação expressas em unidades de Gy.cm^2^, cGy.cm^2^ e mGy.cm^2^ foram convertidas e registradas em μGy.m^2^. Além disso, a razão DAP/peso (μGy.m^2^/kg) foi analisada entre as categorias de cateterismo para possíveis comparações e padronização das doses de radiação. Procedimentos sem dados relacionados à dose de radiação, ou dose de radiação registrada em diferentes unidades, foram excluídos do estudo.

Os procedimentos de cateterismo terapêutico foram divididos em 10 categorias. A exposição à radiação foi avaliada após a categorização dos pacientes em subgrupos de idade (<1 ano; 1 a 4 anos; 5 a 9 anos; 10 a 14 anos; ≥ 15 anos) e peso (até 7 kg; até 15 kg; até 28 kg; > 28 kg). Os dados referentes a: DAP, razão DAP/peso, idade, peso, tempo fluoroscópico e produto peso-fluoroscópico não eram normalmente distribuídos e, portanto, eram descritos como medianas (intervalo interquartil).

### Análise Estatística

Todos os dados foram analisados usando o SPSS (IBM, SPSS *Statistics* , Versão 22.0. Armonk, NY: IBM Corp).

O método Kolmogorov Smirnov foi o teste estatístico utilizado para verificar a normalidade dos dados. As variáveis contínuas não apresentaram distribuição normal após a aplicação do teste de Kolmogorov-Smirnov. As variáveis quantitativas não normalmente distribuídas são apresentadas como medianas (intervalo interquartil). As variáveis categóricas são apresentadas como frequências absolutas (n). As associações entre variáveis contínuas foram avaliadas pelo teste do coeficiente de correlação de Spearman (r_s_). A relação entre variáveis quantitativas contínuas não paramétricas e duas variáveis categóricas foi avaliada pelo teste U de Mann-Whitney. A relação entre variável quantitativa contínua não paramétrica e mais de duas variáveis categóricas foi avaliada pelo teste de Kruskal-Wallis. O valor de p < 0,05 foi considerado estatisticamente significante.

## Resultados

A análise incluiu um total de 1.311 pacientes com idade <18 anos de 16 centros participantes do estudo CHAIN. Entre esses, 206 pacientes não tinham registro de doses de radiação e foram excluídos. Dos 1.026 pacientes restantes com doses registradas de radiação, 597 foram excluídos porque suas doses não foram registradas como DAP. Isso resultou em um total de 429 pacientes participantes (56,4% do sexo masculino) de seis centros. Após a aplicação desses critérios de exclusão, três dos seis centros contribuíram com 90% dos dados dos pacientes.

Os dados demográficos e as características da população e grupos de procedimentos estão descritos na Tabela 1 .

**Tabela 1:** – Dados demográficos e características dos procedimentos

		Procedimentos diagnósticos	Procedimentos intervencionistas	p
Pacientes	429	151	278	
Idade (meses)	50,1 (10, 102,9)	38,8 (13,6, 104,5)	53 (9,2, 102,6)	0,892
Peso (kg)	15 (7,2, 28)	12 (7,2, 27)	16 (7,1, 29,5)	0,466
Tempo de procedimento (min)	40 (27,5, 57)	35 (25, 50)	45 (30, 60)	0,000
Tempo fluoroscópico (min)	9 (5, 15)	8 (4, 13)	9 (5,7, 16)	0,003
Peso x tempo fluoroscópico (kg.min)	114 (54,5, 250)	90 (45, 224)	128 (60, 277)	0,006
DAP (uGy. m^2^)	742 (288,8, 1.791,8)	715,2 (230, 1.534,9)	751,5 (315,4, 2.095,2)	0,14
DAP/peso (uGy.m^2^/kg)	57,2 (28, 124,9)	57 (23,3, 110,5)	57 (30,5, 139,5)	0,137

*Os resultados são descritos em medianas e intervalo interquartil (percentil 25, 75). DAP: produto dose-área. Significância estatística quando p ≤ 0,05.*

O DAP mediano na população estudada foi de 742,2 (288,8, 1.791,5) μGy.m^2^. Os procedimentos intervencionistas apresentaram DAP mediano mais alto que os procedimentos de diagnóstico: 751 (315, 2.095) versus 715 (230, 1.535) μGy.m^2^, respectivamente. Não foram observadas diferenças na razão DAP/peso entre os procedimentos diagnóstico e terapêutico: 57 (23, 110) versus 57 (30, 139), respectivamente.

**Figura 1:**
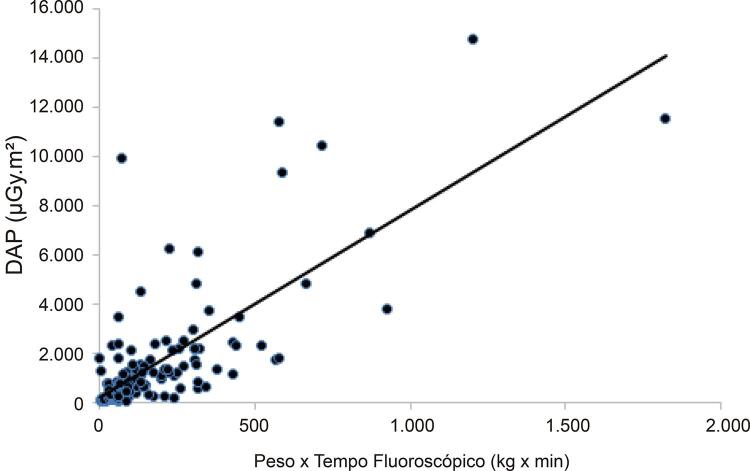
– *Gráfico de dispersão mostra a relação entre produto dose-área (DAP) e peso-produto tempo fluoroscópico em pacientes pediátricos submetidos a cateterismo cardíaco diagnóstico (r = 0,75).*

Verificou-se que o DAP apresenta uma boa correlação com o produto do tempo peso-fluoroscópico (r_s_ = 0,66), e esse padrão de correlação também foi observado quando os procedimentos diagnósticos e terapêuticos foram analisados separadamente (r_s_ = 0,56 e r_s_ = 0,72, respectivamente) ( Figuras 1 e 2 ). Pacientes categorizados em subgrupos de peso demonstraram doses mais altas de radiação (DAP) em procedimentos terapêuticos do que em diagnósticos (p = 0,001). Quando os pacientes foram categorizados em subgrupos etários, observou-se diferença significativa nas doses de radiação entre procedimentos diagnósticos e terapêuticos, mas apenas em pacientes com idade > 15 anos (p = 0,004; Tabela 2 ).

**Figura 2:**
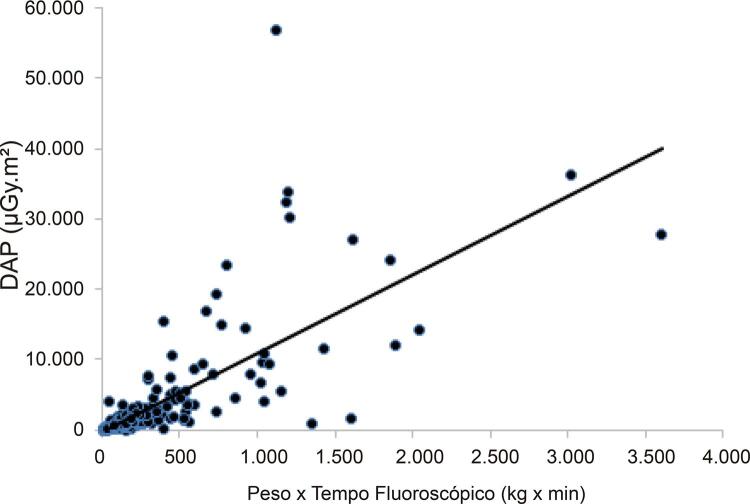
– *O gráfico de dispersão mostra a relação entre o produto dose-área (DAP) e o produto tempo fluoroscópico-peso em pacientes pediátricos submetidos a cateterismo cardíaco terapêutico (r = 0,74).*

**Tabela 2:** – Produto dose-área (DAP; uGy.m2) dos cateterismos diagnósticos e terapêuticos estratificados por faixas etárias

	Tipo de cateterismo		

Faixa etária	Diagnóstico	Terapêutico	p
< 1 ano	n= 36 303,8 (172; 754)	n=78 250,7 (138,6; 570,7)	0,25
1-4 anos	n= 50 524,8 (194,3; 1.038,7)	n=73 602,7 (409,5; 1.329,3)	0,06
5-9 anos	n=75 1.340 (428,9; 2.175,9)	n=71 1.189,7 (491,7; 2.125,4)	0,82
10-14 anos	n= 119 1.739,6 (773,7; 4.524,5)	n=38 2.765 (1.385,3; 8.399,4)	0,08
> 15 anos	n= 11 2.182,2 (295,1; 3.735,7)	n= 18 11.723,5 (5.493,5; 28.357,2)	0,004

*Valores de DAP descritos em medianas e intervalos interquartis (percentil 25, 75). n: número absoluto de pacientes. Significância estatística quando p ≤ 0,05.*

A Tabela 3 destaca os diferentes procedimentos, tempos fluoroscópicos e as proporções correspondentes de DAP/peso. As maiores razões de DAP/peso foram observadas para implante valvar pulmonar percutâneo (Melody), fechamento de defeitos do septo ventricular (DSV) e angioplastia com balão ou *stent* na via de saída do ventrículo direito (VSVD) ou artéria pulmonar (PA), com médias de 273,8, 169,2 e 155,9, respectivamente. Além disso, houve uma diferença significativa entre os subgrupos do procedimento de intervenção e as proporções DAP/peso (p<0,001).

**Tabela 3:** – Tempo fluoroscópico e produto dose-área normalizado indexado ao peso corporal (DAP/peso; uGy.m2/kg) estratificados por tipos de procedimentos

	Pacientes	%	Tempo fluoroscópico	DAP/peso (uGy.m^2^/kg)
Diagnóstico	151	37,3	8 (4; 13)	57,2 (23; 110,5)
Valvoplastia pulmonar	44	10,9	10 (7; 15)	51,8 (35; 93)
Valvoplastia aórtica	20	4,9	9 (7; 13)	59,8 (29,1; 125,9)
Oclusão de PDA	56	13,8	6 (5; 9)	41,9 (27,6; 71,4)
Fechamento do dispositivo DSA/FOP	52	12,8	5 (4; 7,7)	25,5 (13,5; 36,2)
Fechamento do dispositivo DSV	6	1,5	20 (10; 44)	170 (71,4; 513,4)
Angioplastia ou *stent* VSVD/AP	35	8,6	17 (11; 27)	155,9 (75,9; 224,5)
Angioplastia aórtica / *Stent* aórtico	32	7,9	11 (6; 16,7)	98,2 (42; 206,6)
*Stent* PDA	6	1,5	9 (8,5; 15,5)	77,2 (58; 126,6)
Implante de válvula Melody	3	0,7	36 (p25 = 34)	273,8 (p25 = 41,9)

*Tempo de fluoroscopia e valores de DAP descritos em medianas e intervalos interquartis (percentil 25, 75) n = número absoluto de pacientes. Significância estatística quando p ≤ 0,05. PDA: ducto arterioso patente; DAS: defeitos do septo atrial; FOP: forame oval patente; DSV: defeito do septo ventricular; VSVD: via de saída do ventrículo direito; AP: artéria pulmonar.*

## Discussão

Nos últimos anos, a complexidade e o número de procedimentos transcateter aumentaram.^4^ Assim, métodos para proteger pacientes e funcionários da exposição cumulativa à radiação ionizante e seus potenciais efeitos são importantes e, portanto, o estabelecimento de dados de referência é crucial.^8^ Atualmente, as principais limitações para estabelecer valores de referência com procedimentos intervencionistas para cardiopatias congênitas são a falta de padronização das unidades de dosagem e medida^9^ e a existência de uma ampla variedade de procedimentos e complexidades, variações de peso e idade, tipos de equipamentos e habilidades médicas. Todos esses fatores contribuem para uma grande heterogeneidade, o que dificulta comparações.^4 , 8^ O *Food and Drug Administration* e a Organização Mundial da Saúde recomendam o registro do DAP e o cálculo de doses efetivas para todos os pacientes submetidos a procedimentos que utilizam radiação.^10^ Com base nessa proposta, avaliaram-se neste momento 429 pacientes com idade <18 anos e registrados no estudo CHAIN. Embora relativamente menor que o número de pacientes relatados em estudos anteriores,^4 , 7 , 8 , 11 , 12^ os resultados da presente análise revelam o potencial do uso da razão DAP/peso como referência para comparação.

A ausência de diferença estatística no DAP entre os procedimentos diagnósticos e terapêuticos no presente estudo pode ser explicada pelos recentes avanços nos procedimentos intervencionistas de baixa complexidade, como o fechamento percutâneo de defeitos do septo atrial (DSA), forame oval patente (PFO), e ducto arterioso patente ( *Patent ductus arteriosus* – PDA), além da valvoplastia pulmonar, que utiliza doses de radiação relativamente baixas. Além disso, os procedimentos de diagnóstico geralmente envolvem pacientes com doenças cardíacas complexas sem um diagnóstico definido, exigindo alto tempo de fluoroscopia.

Durante a análise do cateterismo diagnóstico e terapêutico, observou-se que o DAP aumentou à medida que a idade aumentou. Quando os dois procedimentos foram comparados com os subgrupos etários, não foram observadas diferenças estatísticas, exceto no grupo com idade > 15 anos, em que a dose de radiação foi significativamente maior nos procedimentos terapêuticos, semelhante ao relatado por Ubeda et al.^13^ Isso provavelmente foi resultado do maior número de procedimentos complexos, como implante valvar percutâneo e angioplastia em pacientes mais velhos.

Os principais procedimentos intervencionistas analisados no presente estudo tiveram medianas de dose comparáveis às relatadas em estudos recentes^3 - 8 , 11 , 13 , 14^ ( *Tabela 3* ), principalmente quando os valores foram comparados pela razão DAP/peso, que padroniza valores crescentes de DAP relacionado às diferenças de peso no mesmo procedimento. A variação da razão DAP/peso entre os diferentes tipos de cateterismo intervencionista foi estatisticamente significante, como demonstrado em outros estudos.^2 , 8 , 11 , 12 , 14^ As maiores doses de radiação foram observadas em implantes valvares pulmonares percutâneos (Melody), fechamento de DSV e angioplastias com balão ou *stent* em VSVD ou AP, como relatado anteriormente.^8 , 11^ As medianas da razão DAP/peso em valvoplastias pulmonares, fechamento de DSV e angioplastias com balão ou *stent* de VSVD ou AP foram semelhantes às obtidas por Kobayashi e Borik et al.^8 , 11^

Em muitos procedimentos no presente estudo, as medianas do DAP foram inferiores às observadas em estudos anteriores.^3 , 4 , 15^ Glatz et al.^15^ avaliaram 2.265 pacientes em um estudo de centro único e obtiveram um DAP mediano significativamente maior que na maioria dos procedimentos estudados, mas o estudo incluiu adultos e pacientes com peso > 65 kg (máximo, 128 kg). Por outro lado, o estudo CHAIN apresentou um peso mediano de 21 kg. O único procedimento relatado por Glatz et al. com uma dose menor que a do presente estudo foi a aortoplastia com balão/ *stent* (DAP de 484 *versus* 1,904 μGy.m^2^ , respectivamente). Ghelani et al. publicaram um estudo realizado de 2009 a 2011 com 2.713 pacientes nos quais o DAP de alguns procedimentos intervencionistas foi avaliado. As medianas relatadas do DAP foram superiores às de outros estudos, incluindo o estudo CHAIN. Esses resultados também podem ser parcialmente justificados pela inclusão de pacientes com idade > 15 anos e adultos, representando aproximadamente 20% da população avaliada. No entanto, neste estudo, a relação DAP/Kg não foi avaliada. Todos esses dados corroboram o conceito de que o uso da razão DAP/peso é uma medida racional para padronizar a avaliação da dose de radiação em uma população pediátrica heterogênea. De acordo com essa linha de pensamento, Cevallos et al publicaram recentemente novos parâmetros de referência para dosagem de radiação na população pediátrica. Diferentemente do estudo anterior do mesmo grupo^4^ , eles avaliaram o DAP/kg estratificado por faixas etárias e tipos de procedimentos, o que permite a comparação com a literatura atual.^12^ Este estudo foi realizado após os esforços de melhoria da qualidade da radiação (MQ) nos diferentes centros envolvidos. Curiosamente, as doses médias encontradas por nosso grupo no presente foram muito semelhantes às relatadas por Cevallos et al. após um programa de MQ ( Tabela 4 ).

**Tabela 4:** – Comparação de nossos dados estratificados por tipo de procedimentos, dados de radiação de procedimentos (CHAIN) com bancos de dados de doses de radiação publicados anteriormente

Procedimentos	Manica, 2018 (CHAIN)		Cevallos, 2017 (C3PO)		Borik, 2015		Kobayashi, 2014 (CCISC)		Onnasch, 2007	

	n	^a^ DAP/peso	n	^b^ DAP/peso	n	^a^ DAP/peso	n	^c^ DAP/peso	n	^c^ DAP/peso
Valvoplastia pulmonar	44	**51,8** (34-92)	258	53 (104-335)	286	**28** (1-345)	342	**56** (152)	-	-
Valvoplastia aórtica	20	**59,8** (29-126)	136	**99** (165-383)	138	**42** (8-211)	138	**80** (127)	-	-
Oclusão de PDA	56	**41,9** (27-71)	443	**37** (72-217)	266	**18** (4-251)	467	**42** (71)	165	**34,5** (37)
Fechamento do dispositivo DSA/FOP	52	**25,5** (13-36)	295	**34** (64-199)	345	**21** (2-367)	568	**41** (71)	259 / 21	**41,9** (50)/ **23** (30)
Fechamento do dispositivo DSV	6	**169,2** (71-513)	-	-	-	-	-	-	32	**130** (175)
Angioplastia ou stent VSVD/AP	35	**155,9** (76-224)	-	-	366	**102** (8-910)	427	**132** (222)	-	-
Angioplastia aórtica	32	**98,2** (42-206)	288	**90** (165-384)	120	**43** (7-447)	182	**66** (107)	-	-
Stent aórtico					52	**80** (13-448)	112	**90** (159)	-	-
Stent PDA	6	**77,2** (58-126)	-	-	-	-	-	-	-	-
Implante de válvula Melody	3	**273,8**	199	**257** (400-671)	38	**191** (60-935)	88	**186** (299)	-	-

*DAP/peso: DAP indexado por peso corporal. Valores de DAP descritos em medianas e faixas interquartis: ^a^(percentil 25, 75); ^bi^(percentil 75, 95); ^c^(Percentil 75). A angioplastia aórtica e o stent são agrupados em CHAIN e C3PO. 75th percentile). n = número absoluto de pacientes. DAP: produto dose–área; PDA: ducto arterioso patente; DAS: defeitos do septo atrial; FO: forame oval; DSV: defeito do septo ventricular; VSVD: via de saída do ventrículo direito; AP: artéria pulmonar; CHAIN: Brasilian registry of Congenital HeArt disease INtervention and angiography; C3PO: Congenital Cardiac Catheterization Project on Outcomes; CCISC: Congenital Cardiovascular Interventional Study Consortium.*

A principal limitação do presente estudo foi a falta de dados de alguns centros participantes, provavelmente devido à ausência de padronização dos dados coletados. Como conseqüência, a amostra estudada foi menor e possivelmente menos heterogênea. Ao mesmo tempo, isso corrobora a hipótese de falta de padronização das medidas de exposição à radiação em populações pediátricas e demonstra que vários centros brasileiros ainda não relatam adequadamente a dose de radiação usada em seus procedimentos. Isso reforça a necessidade de conscientização das instituições em relação a um controle apropriado e a um programa de garantia de qualidade bem desenvolvido para segurança contra radiação. Além disso, em algumas análises o número de pacientes avaliados foi pequeno e portanto a análise estatística não foi possível, por como exemplo, no implante valvar pulmonar percutâneo. No entanto, as doses de radiação que esses pacientes receberam foram semelhantes às citadas na literatura.

## Conclusão

A dose de radiação aumenta com a idade do paciente e a complexidade do procedimento. No presente estudo, as doses de radiação observadas foram semelhantes às de outros estudos relatados. As doses de radiação nesses procedimentos devem servir de referência para outras instituições para o controle apropriado da exposição à radiação de pacientes e funcionários.

A proporção DAP/peso parece ser a medida de radiação mais útil e aplicável para o estabelecimento de uma dose de referência para a população pediátrica, uma vez que permite a eliminação de categorias etárias e engloba o amplo espectro de tamanhos corporais. Dessa forma, novos estudos utilizando a razão DAP/peso são importantes para o desenvolvimento de doses de referência em procedimentos hemodinâmicos e para a avaliação de estratégias, visando reduzir a exposição à radiação de pacientes e funcionários.
