# Supplementary material for: Padronizando a Exposição à Radiação durante o Cateterismo Cardíaco em Crianças com Cardiopatia Congênita: Dados de um Registro Multicêntrico Brasileiro
Source: Arq Bras Cardiol. 2020 Dec 1;115(6):1154–661. [Article in Portuguese] doi: 10.36660/abc.20190012 (PMC8133734; doi:10.36660/abc.20190012)
Supplement: Translation

# Translation: Standardizing Radiation Exposure during Cardiac Catheterization in Children with Congenital Heart Disease: Data from a Multicenter Brazilian Registry

**Authors:** João Luiz Manica, Vanessa Oliveira Duarte, Marcelo Ribeiro, Adam Hartley, Ricardo Petraco, Carlos Pedra, Raul Rossi

**Affiliations:** 1 Brazil Instituto de Cardiologia / Fundação Universitária de Cardiologia (IC/FUC), Porto Alegre, RS - Brazil; 2 Brazil Hospital do Coração, São Paulo, SP - Brazil; 3 United Kingdom National Heart and Lung Institute, Imperial College London, Hammersmith Hospital, London - United Kingdom

**Keywords:** Diagnostic,Imaging/methods, Radiation Exposure Pathways, Heart Defects, Congenital, Cardiac Catheterization/methods, Child

## Abstract

**Background:**

In recent years the increasing number of interventional procedures has resulted in growing concerns regarding radiation exposure for patients and staff. The evaluation of radiation exposure in children is difficult due to the great variability in body weight. Therefore, reference levels of radiation are not well defined for this population.

**Objectives:**

To study and validate the ratio of dose-area product (DAP) to patient weight as a reference measurement of radiation for hemodynamic congenital heart disease procedures in children.

**Methods:**

This observational multicenter study uses data obtained from a Brazilian registry of cardiac catheterization for congenital heart disease from March 2013 to June 2014. Inclusion criteria were all patients aged <18 years old undergoing hemodynamic procedures for congenital heart disease, with recorded DAP doses. P-value < 0.05 was considered as statistically significant.

**Results:**

This study evaluated 429 patients with median age and weight of 50 (10, 103) months and 15 (7, 28) kg, respectively. Median DAP was 742.2 (288.8, 1,791.5) μGy.m2. There was a good correlation between DAP and weight-fluoroscopic time product(rs=0.66). No statistically significant difference was observed in DAP/weight ratio between therapeutic and diagnostic procedures. There was a wide variation in the DAP/weight ratio among the therapeutic procedures (p<0.001).

**Conclusions:**

The DAP/weight ratio is the simplest and most applicable measurement to evaluate radiation exposure in a pediatric population. Although there is limited literature available, the doses obtained in the present study were similar to those previously found. Ongoing research is important to evaluate the impact of strategies to reduce radiation exposure in this population (Arq Bras Cardiol. 2020; [online].ahead print, PP.0-0)

## Introduction

Over the last 20 years, cardiac catheterization has not only been used as a diagnostic examination for congenital heart diseases, but has also played an important role in palliative and definitive treatments of more than 50% of patients with congenital heart diseases.^1^ During this period, the complexity, duration, and number of percutaneous procedures have increased, along with a consequent increase in the exposure of patients to ionizing radiation.^2 - 4^

Children are highly sensitive to ionizing radiation, due to their higher proportion of actively dividing cells and the large fraction of exposed body area.^2^ Thus, there is a great concern about the cumulative effects, particularly the high risk of malignancy caused by long-term chromosomal damage, with reports demonstrating that children are up to ten times more susceptible to the development of cancer by radiation exposure than adults.^5 , 6^ In addition, the effective radiation dose is higher for children, resulting in a higher radiation dose for surrounding organs when an area of interest is being assessed.

There are limited studies on radiation doses emitted during interventions in children with congenital heart disease.^3 , 7^ To achieve a reduction in the radiation dose, it is essential to establish reference doses that allow comparisons between procedures.^4^ However, it is difficult to evaluate the radiation exposure in a pediatric population due to the differences in the complexity of procedures, age and weight of the patients, as well as in the types of equipment used.^8^ Moreover, the calculation of the estimated effective radiation dose is complex. Currently, the total radiation dose (total air kerma) and total dose-area product (DAP; air kerma-area product), which is a better estimator of stochastic (long-term radiation effects and risk of malignancy) and cumulative effects of exposure, are used as indicators of a cumulative radiation dose to the skin.

Recently, Chida et al.^2^ and Kobayashi et al.^8^ observed a correlation between DAP and weight as a reference radiation dose in children. They concluded that the radiation dose tends to vary proportionally to patient size. In this context, the present study aims to evaluate the DAP/weight ratio as a reference for radiation exposure in pediatric cardiac catheterization procedures performed in Brazil.

## Materials and Methods

### Study Design and Population

This is a cross-sectional observational study in which patients aged <18 years old and participating in the Congenital Heart Disease Intervention and Angiography (CHAIN) registry, a Brazilian registry of cardiac catheterization for congenital heart disease, were evaluated after undergoing a diagnostic or interventional procedure between March 5^th^, 2013 and June 30^th^, 2014.

The CHAIN registry is a national multicenter prospective study, coordinated by the Teaching and Research Institute of *Hospital do Coração* , together with the Ministry of Health and the Brazilian Society of Hemodynamics and Interventional Cardiology. The main objective was to gather prospective data and create a national registry of catheterization of patients with congenital heart diseases, as well as to propose a comprehensive analysis of the current status and devise effective action measures for public health in Brazil.

Patients who underwent electrophysiological procedures or those in whom vascular access was achieved using hybrid procedures were excluded from the study. Patients who underwent more than one catheterization on different dates were considered as distinct patients in each procedure and were included in the overall statistics as well as in the group of each specific procedure. Patients who underwent more than one intervention using the same procedure were classified according to the most complex procedure.

### Analyzed Variables

Demographic characteristics of patients, such as age, gender, weight, body surface, type of heart disease, and residual lesions, were obtained from the CHAIN registry, in addition to data regarding the hemodynamic procedure performed, including fluoroscopic time and radiation exposure dose. DAP, which represents the radiation dose measured in the air in relation to the distance from the X-ray tube multiplied by the X-ray beam area at this distance, was expressed in μGy.m^2^. Radiation measurements expressed in units of Gy.cm^2^, cGy.cm^2^, and mGy.cm^2^were converted and recorded in μGy.m^2^. Moreover, the DAP/weight ratio (μGy.m^2^/kg) was analyzed among the catheterization categories for possible comparisons and standardization of radiation doses. Procedures lacking data related to radiation dose, or radiation dose recorded in different units, were excluded from the study.

Therapeutic catheterization procedures were divided into 10 categories. Radiation exposure was evaluated after the patients were categorized into age (<1 year; 1–4 years; 5–9 years; 10–14 years, and ≥15 years) and weight (up to7 kg; up to15 kg; up to 28 kg; >28 kg) subgroups. Data regarding DAP, DAP/weight ratio, age, weight, fluoroscopic time, and weight–fluoroscopic time product were not normally distributed, and were, therefore, described as medians (interquartile range).

### Statistical Analysis

All data were analyzed using SPSS (IBM, SPSS Statistics, Version 22.0. Armonk, NY: IBM Corp).

The Kolmogorov Smirnov method was the statistical test used to verify the normality of the data. Continuous variables did not present normal distribution after the Kolmogorov-Smirnov test was applied. Non-normally distributed quantitative variables are presented as medians (interquartile range). Categorical variables are presented as absolute frequencies (n). Associations between continuous variables were evaluated using the Spearman correlation coefficient test (r_s_). The relationship between non-parametric continuous quantitative and two categorical variables were assessed using the Mann-Whitney U test. The relationship between non-parametric continuous quantitative and more than two categorical variables was assessed using the Kruskal-Wallis test. P-value < 0.05 was considered as statistically significant.

## Results

A total of 1,311 patients aged <18 years old from 16 participating centers participating in the CHAIN study were included in the analysis. Among those, 206 patients had no records on radiation doses and were excluded. Of the remaining 1,026 patients with recorded radiation doses, 597 were excluded as their doses were not recorded as DAP. This resulted in a total of 429 participating patients (56.4% male) from six centers. After these exclusion criteria were applied, three out of the six centers contributed 90% of patient data.

Demographic data and the characteristics of the population and procedure groups are described in Table 1 .

**Table 1:** – Demographic data and characteristics of the procedures

		Diagnostic procedures	Interventional procedures	p
Patients	429	151	278	
Age (months)	50.1 (10; 102.9)	38.8 (13.6; 104.5)	53 (9.2; 102.6)	0.892
Weight (kg)	15 (7.2; 28)	12 (7.2; 27)	16 (7.1; 29.5)	0.466
Procedure time (min)	40 (27.5; 57)	35 (25; 50)	45 (30; 60)	0.000
Fluoroscopic time (min)	9 (5; 15)	8 (4; 13)	9 (5.7; 16)	0.003
Weight x fluoroscopic time (kg.min)	114 (54.5; 250)	90 (45; 224)	128 (60; 277)	0.006
DAP (uGy. m^2^)	742 (288.8; 1,791.8)	715.2 (230; 1,534.9)	751.5 (315.4; 2,095.2)	0.14
DAP/weight (uGy.m^2^/kg)	57.2 (28; 124.9)	57 (23.3; 110.5)	57 (30.5; 139.5)	0.137

*Results are described in medians and interquartile range (25^th^, 75^th^percentile). DAP: dose-area product. Statistical significance when p ≤ 0.05.*

The median DAP in the studied population was 742.2 (288.8, 1,791.5) μGy.m^2^. Interventional procedures had higher median DAP than diagnostic ones: 751 (315, 2,095) *versus* 715 (230, 1,535) μGy.m^2^, respectively. No differences were observed in the DAP/weight ratio between diagnostic and therapeutic procedures: 57 (23, 110) *versus* 57 (30, 139) respectively.

DAP was found to have a good correlation with the weight–fluoroscopic time product (r_s_= 0.66), and this correlation pattern was also observed when diagnostic and therapeutic procedures were separately analyzed (r_s_= 0.56 and r_s_= 0.72, respectively) ( Figures 1 and 2 ). Patients categorized into weight subgroups demonstrated higher radiation doses (DAP) in therapeutic than in diagnostic procedures (p= 0.001). When patients were categorized into age subgroups, a significant difference in radiation doses was observed between diagnostic and therapeutic procedures, but only in patients aged >15 years (p=0.004; Table 2 ).

**Figure 1:** – *Scatterplot shows relationship between dose–area product (DAP) and weight– fluoroscopic time product in pediatric patients who underwent diagnostic cardiac catheterization (r = 0.75).*

**Figure 2:** – *Scatterplot shows relationship between dose–area product (DAP) and weight– fluoroscopic time product in pediatric patients who underwent diagnostic cardiac catheterization (r = 0.75).*

**Table 2:** – Dose-area product (DAP; uGy.m2) of diagnostic and therapeutic catheterizations stratified by age groups

	Type of Catheterization		

Age group	Diagnostic	Therapeutic	p
< 1 year	n= 36 303.8 (172; 754)	n=78 250.7 (138.6; 570.7)	0.25
1-4 years	n= 50 524.8 (194.3; 1,038.7)	n=73 602.7 (409.5; 1,329.3)	0.06
5-9 years	n=75 1,340 (428.9; 2,175.9)	n=71 1,189.7 (491.7; 2,125.4)	0.82
10-14 years	n= 119 1,739.6 (773.7; 4,524.5)	n=38 2,765 (1,385.3; 8,399.4)	0.08
> 15 years	n= 11 2,182.2 (295.1; 3,735.7)	n= 18 11,723.5 (5,493.5; 28,357.2)	0.004

*DAP values described in medians and interquartile ranges (25^th^, 75^th^percentile). n: absolute number of patients. Statistical significance when p ≤ 0.05.*

Table 3 highlights the different procedures, fluoroscopic times, and corresponding DAP/weight ratios. The highest DAP/weight ratios were observed for percutaneous pulmonary valve implantation (Melody), closure of ventricular septal defects (VSD), and balloon or stent angioplasty in the right ventricular outflow tract (RVOT) or pulmonary artery (PA), with means of 273.8, 169.2, and 155.9, respectively. In addition, there was a significant difference between intervention procedure subgroups and DAP/weight ratios (p<0.001).

**Table 3:** – Fluoroscopic time and normalized dose–area product indexed to body weight(DAP/weight; uGy.m2/kg) stratified by procedure types

	Patients	%	Fluoroscopy time	DAP/weight (uGy.m^2^/kg)
Diagnostic	151	37.3	8 (4; 13)	57.2 (23; 110.5)
Pulmonary valvuloplasty	44	10.9	10 (7; 15)	51.8 (35; 93)
Aortic valvuloplasty	20	4.9	9 (7; 13)	59.8 (29.1; 125.9)
PDA occlusion	56	13.8	6 (5; 9)	41.9 (27.6; 71.4)
ASD/PFO device closure	52	12.8	5 (4; 7.7)	25.5 (13.5; 36.2)
VSD device closure	6	1.5	20 (10; 44)	170 (71.4; 513.4)
RVOT/PA angioplasty or stent	35	8.6	17 (11; 27)	155.9 (75.9; 224.5)
Aortic angioplasty/ Aortic stent	32	7.9	11 (6; 16.7)	98.2 (42; 206.6)
PDA Stent	6	1.5	9 (8.5; 15.5)	77.2 (58; 126.6)
Melody valve implant	3	0.7	36 (p25 = 34)	273.8 (p25 = 41.9)

*Fluoroscopy time and DAP values described in medians and interquartile ranges (25^th^, 75^th^percentile). n: absolute number of patients. Statistical significance when p ≤ 0.05. PDA: patent arterial duct; ASD: atrial septal defect; PFO: patent foramen ovale; VSD: ventricular septal defect; RVOT: right ventricular outflow tract; PA: pulmonary artery.*

## Discussion

In recent years, the complexity and number of transcatheter procedures have increased.^4^ Thus, methods to protect patients and staff from cumulative exposure to ionizing radiation and its potential effects are important and, therefore, establishing reference data is crucial.^8^ Currently, the major limitations for setting reference values with interventional procedures for congenital heart diseases are the lack of standardization of dosage and measurement units,^9^ as well as the existence of a wide variety of procedures and complexities, weight and age variations, types of equipment and medical abilities. All these factors contribute to a great heterogeneity, which makes comparisons difficult.^4 , 8^ The Food and Drug Administration and the World Health Organization recommend recording DAP and calculating effective doses for all patients undergoing procedures utilizing radiation.^10^ Based on this proposal, a total of 429 patients aged <18 years and registered in the CHAIN study were evaluated in the present study. Although relatively smaller than the number of patients reported in previous studies,^4 , 7 , 8 , 11 , 12^ the results from the present analysis reveal the potential of using the DAP/weight ratio as a reference for comparison.

The absence of a statistical difference in DAP between diagnostic and therapeutic procedures in the present study can be explained by recent advances in low-complexity interventional procedures, such as the percutaneous closure of atrial septal defects (ASD), patent foramen ovale (PFO), and patent arterial duct (PDA), in addition to pulmonary valvuloplasty, which use relatively low radiation doses. Furthermore, diagnostic procedures often involve patients with complex cardiac diseases without a defined diagnosis, requiring high fluoroscopy times.

During the analysis of diagnostic and therapeutic catheterization, it was observed that DAP increased as age increased. When the two procedures were compared with age subgroups, no statistical differences were observed, except in the group aged >15 years, in which the radiation dose was significantly higher in therapeutic procedures, similar to that reported by Ubedaet al.,^13^ This was likely a result of a higher number of complex procedures, such as percutaneous valve implantation and angioplasty in older patients.

The main interventional procedures analyzed in the present study had dose medians comparable to those reported in recent studies^3 - 8 , 11 , 13 , 14^ ( Table 3 ), particularly when values were compared using the DAP/weight ratio, which standardizes increasing values of DAP related to weight differences in the same procedure. The variation of the DAP/weight ratio between the different types of interventional catheterization was statistically significant, as demonstrated in other studies.^2 , 8 , 11 , 12 , 14^ The highest doses of radiation were observed in percutaneous pulmonary valve implantations (Melody), closures of VSD, and balloon or stent angioplasties in RVOT or PA, as reported previously.^8 , 11^ The medians of the DAP/weight ratio in pulmonary valvuloplasties, closures of VSD, and balloon or stent angioplasties of RVOT or PA were similar to those obtained by Kobayashi and Borik et al.,^8 , 11^

In many procedures in the present study, DAP medians were lower than those observed in previous studies.^3 , 4 , 15^ Glatz et al.,^15^ evaluated 2,265 patients in a single-center study and obtained a median DAP significantly higher than in most procedures studied, including adults and patients who weighed >65 kg (maximum, 128 kg). In contrast, the CHAIN study presented a median weight of 21 kg. The only procedure reported by Glatz et al. with a lower dose than those of the present study was the balloon/stent aortoplasty (DAP of 484 *versus* 1,904 μGy.m^2^ , respectively). Ghelani et al. published a study conducted from 2009 to 2011 with 2,713 patients in which the DAP of some interventional procedures was evaluated. The reported DAP medians were higher than those of other studies, including the CHAIN study. These results can also be partially justified by the inclusion of patients aged >15 years and adults, representing approximately 20% of the evaluated population. However, in this study, DAP/Kg was not evaluated. All these data corroborate the concept that the use of the DAP/weight ratio is a rational measure to standardize the evaluation of radiation dose in a heterogeneous pediatric population. In accordance with this line of thought, Cevallos and the C3PO group recently published new benchmarks for radiation dosage in the pediatric population. Differently from the previous study by the same group^4^ , they assessed DAP/Kg stratified by age groups and procedure types, which allows for comparison with the current literature.^12^ This study was performed after radiation quality improvements (QI) efforts in the different centers involved. Interestingly, the mean doses found by our group in the present were very similar to those reported by Cevallos et al. after a QI program ( Table 4 ).

**Table 4:** – Comparison of our data stratified by procedures type procedures radiation data (CHAIN) with previously published radiation dose databases

Procedures	Manica, 2018 (CHAIN)		Cevallos, 2017 (C3PO)		Borik, 2015		Kobayashi, 2014 (CCISC)		Onnasch, 2007	

	n	^a^DAP/w	n	^b^DAP/w	n	^a^DAP/w	n	^c^DAP/w	n	^c^DAP/w
Pulmonary valvuloplasty	44	51.8 (34-92)	258	53 (104-335)	286	28 (1-345)	342	56 (152)	-	-
Aortic valvuloplasty	20	59.8 (29-126)	136	99 (165-383)	138	42 (8-211)	138	80 (127)	-	-
PDA occlusion	56	41.9 (27-71)	443	37 (72-217)	266	18 (4-251)	467	42 (71)	165	34.5 (37)
ASD/PFO device closure	52	25.5 (13-36)	295	34 (64-199)	345	21 (2-367)	568	41 (71)	259 / 21	41.9 (50)/ 23 (30)
VSD device closure	6	169.2 (71-513)	-	-	-	-	-	-	32	130 (175)
RVOT/PA angioplasty or stent	35	155.9 (76-224)	-	-	366	102 (8-910)	427	132 (222)	-	-
Aortic angioplasty	32	98.2 (42-206)	288	90 (165-384)	120	43 (7-447)	182	66 (107)	-	-
Aortic stent					52	80 (13-448)	112	90 (159)	-	-
PDA Stent	6	77.2 (58-126)	-	-	-	-	-	-	-	-
Melody valve implant	3	273.8	199	257 (400-671)	38	191 (60-935)	88	186 (299)	-	-

*DAP/w: DAP indexed by body weight. DAP values described in medians and interquartile ranges:^a^(25^th^, 75^th^percentile);^b^(75^th^, 95^th^percentile);^c^(75^th^percentile). Aortic angioplasty and stenting are grouped together in CHAIN and C3PO. n: absolute number of patients; DAP: dose–area product; PDA: patent arterial duct; ASD: atrial septal defect; FO: foramen ovale; VSD: ventricular septal defect; RVOT: right ventricular outflow tract; PA: pulmonary artery; CHAIN: Brazilian registry of Congenital HeArt disease INtervention and angiography; C3PO: Congenital Cardiac Catheterization Project on Outcomes; CCISC: Congenital Cardiovascular Interventional Study Consortium.*

The main limitation of the present study was the lack of data from some participating centers, probably due to the absence of standardization of the collected data. As a consequence, the studied sample was smaller and possibly less heterogeneous. At the same time, this corroborates the hypothesis of a lack of standardization of radiation exposure measurements in pediatric populations and demonstrates that a number of Brazilian centers do not yet properly report the radiation dose used in their procedures. This reinforces the need for awareness of institutions with regard to an appropriate control and a well-developed quality assurance program for radiation safety. Moreover, in some analyses, the number of patients evaluated was small and thus a statistical analysis was not possible, for example, percutaneous pulmonary valve implantation. Nevertheless, the radiation doses these patients received were similar to those cited in the literature.

## Conclusions

Radiation dose increases with patient age and the complexity of the procedure. In the present study, the radiation doses observed were similar to those from other reported studies. The radiation doses in these procedures should serve as a benchmark for other institutions for appropriate control of radiation exposure of patients and staff.

The DAP/weight ratio appears to be the most useful and applicable measurement of radiation for the establishment of a reference dose for the pediatric population, given that it allows the elimination of age categories and encompasses the broad spectrum of body sizes. As such, new studies using the DAP/weight ratio are important for the development of reference doses in hemodynamic procedures and for the evaluation of strategies aiming to reduce radiation exposure of patients and staff.
